# Supplementary material for: Disruption of prepulse inhibition is associated with compulsive behavior severity and nucleus accumbens dopamine receptor changes in Sapap3 knockout mice
Source: Sci Rep. 2021 May 3;11:9442. doi: 10.1038/s41598-021-88769-5 (PMC8093235; doi:10.1038/s41598-021-88769-5)
Supplement: Supplementary file 1 — Supplementary Information [file 41598_2021_88769_MOESM1_ESM.pdf]

Disruption of prepulse inhibition is associated with severity of compulsive behavior and nucleus accumbens dopamine receptor changes in Sapap3 knockout mice

Elizabeth E Manning[1]; Abigail Y Wang[1]; Linda M Saikali[1]; Anna S Winner[1]; Susanne E Ahmari*[1]

[1]Department of Psychiatry, Translational Neuroscience Program, Center for Neuroscience Program, Center for Neural Basis of Cognition, University of Pittsburgh

Results

PPI and startle in Sapap3-KOs with and without lesions

%PPI increased with increasing prepulse intensity similarly in WT and KO mice (main effect PP, $F_{(2,56)}$=87.4, p<0.0001; main effect genotype: p=0.14, PP x genotype interaction p=0.86; Figure S1A). Startle amplitude did not differ between the groups during the trial blocks used to calculate PPI (2 and 3) (main effect of group, p=0.43; Figure S1B). However, there was a trend for differences in startle habituation between the groups (Figure S1C, group x block interaction p=0.06). KO-L showed a trend for reduced startle in block 1 (vs KO-NL and WT, p<0.06, t_{108}>2.3) and stable startle across the 4 blocks, whereas WT and KO-NL showed typical startle habituation. The amplitude of movement detected during trials when no stimulus was presented was reduced in both KO groups relative to WT (Figure S1D, $F_{(2,27)}$=8.6, p=0.001). Bodyweight

was also reduced in KOs relative to WT, although this effect was most pronounced in KO-L, which were significantly lighter than both WT and KO-NL (Figure S1E, $F_{(2,27)}$=28.5, p<0.0001).

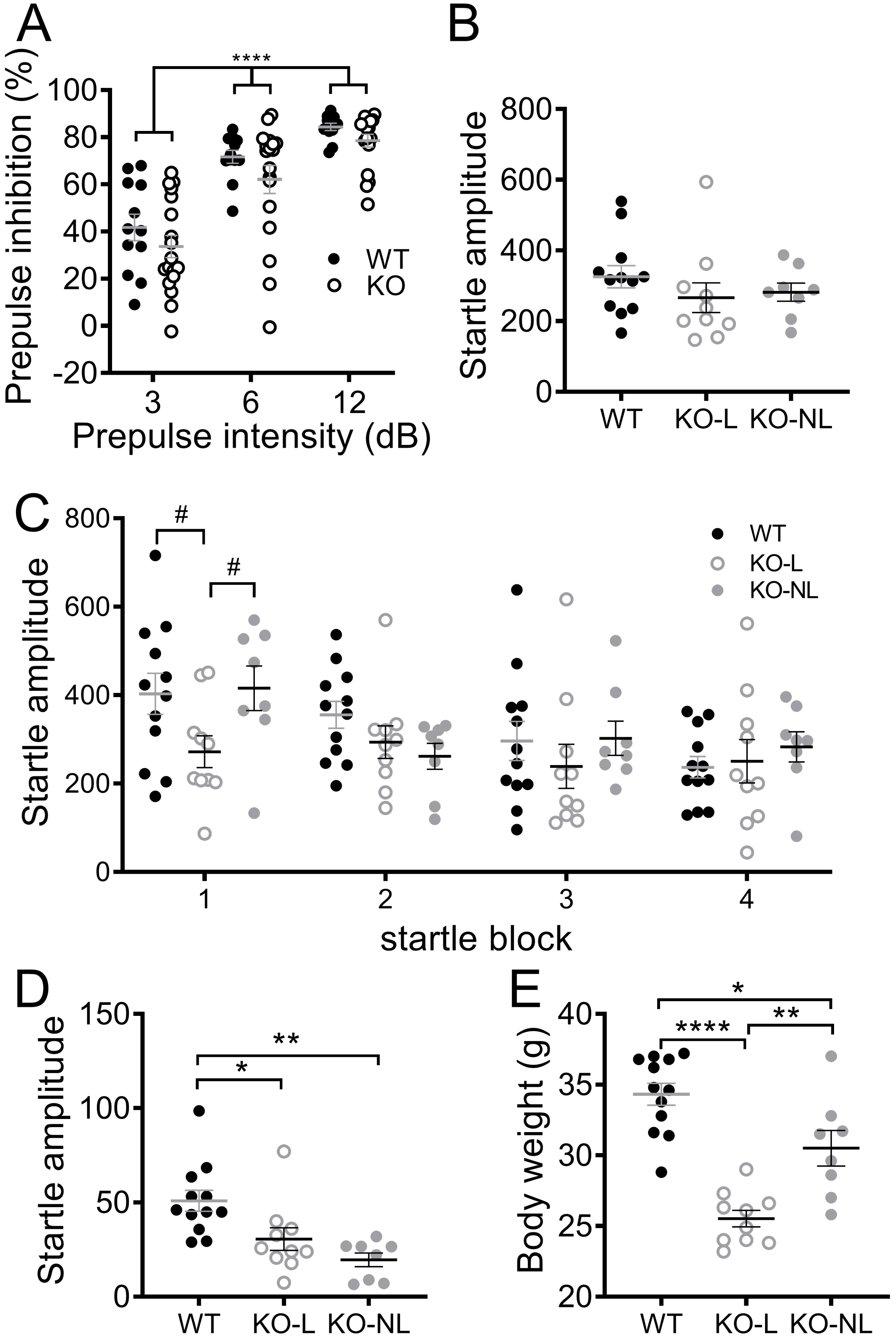

Figure S1. PPI and startle in Sapap3-KOs with and without lesions. **A)** PPI increases with increasing prepulse magnitude to a similar extent in WT and KO mice. **B)** Startle amplitude during 120dB pulse only trials in blocks 2/3 (used to calculate %PPI) does not differ between WT, KO-L and KO-NL. **C)** A trend was observed towards altered habituation to startling stimulus (120dB pulse only trials) between groups across 4 blocks of testing (p=0.058). Post-hoc tests demonstrated that this trend was driven by the KO-L group, which showed reduced startle during the first block relative to the other groups ($t_{(108)}>2.3$, p<0.06), and an attenuation of habituation across the subsequent blocks relative to WT and KO-NL (block 1 vs block 4: KO-L, p>0.99; KO-NL $t_{(81)}=2.7$, p=0.045; WT $t_{(81)}=4.2$, p= 0004). **D)** Movement detected during "no stimulus" trials was reduced in KO-L and KO-NL relative to WT (KO-L vs WT $t_{(27)}=2.8$, p=0.03, KO-NL vs WT $t_{(27)}=4.0$, p=0.0014), but did not differ between KO-L and KO-NL (p=0.57). **E)** Body weight was reduced in KO-L and KO-NL relative to WT (KO-L vs WT $t_{(27)}=7.5$, p<0.001, KO-NL vs WT $t_{(27)}=3.0$, p=0.014). KO-L also showed lower body weight relative to KO-NL ($t_{(27)}=3.9$, p=0.0019). In panel A, * indicates main effect of prepulse intensity, in panel C-E */# indicates results of Bonferroni post-hoc tests. **** p<0.0001; **p<0.01, *p<0.05, # 0.1>p>0.05. n=14 WT, 18 KO (10 KO-L, 8 KO-NL). KO= knockout, KO-L= KOs with lesions, KO-NL= KOs without lesions, WT= wild-type.

Unbiased clustering of PPI and grooming in Sapap3-KO mice

To further examine behavioral heterogeneity in Sapap3-KOs, unbiased clustering was performed to determine whether distinct phenotype groups emerged (Figure S2). A three-cluster solution identified 2 clusters with high grooming (one-way ANOVA grooming: $F_{(2, 15)}=82.0$, p<0.0001), one with impaired PPI (one-way ANOVA PPI: $F_{(2, 15)}= 24.3$, p<0.0001; cluster 1: n=6, all KO-L) and one with relatively intact PPI (cluster 2: n=5, including 1 KO-NL). A third cluster of unimpaired mice was also identified (cluster 3 n=7, all KO-NL).

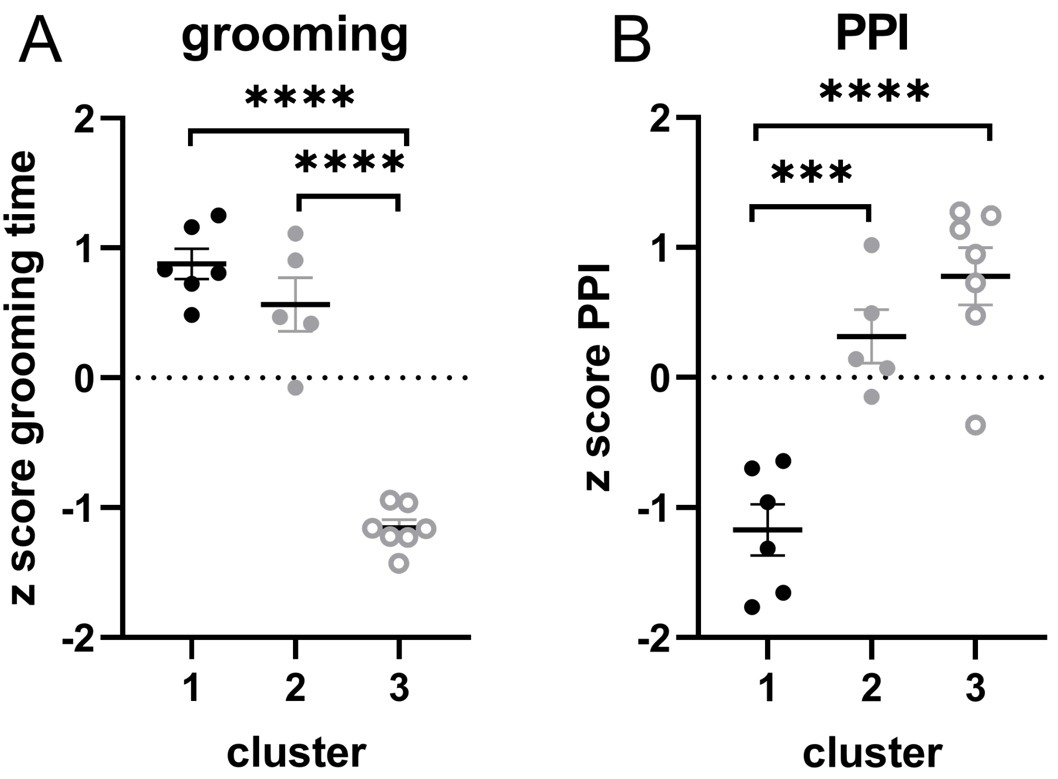

Figure S2: Unbiased clustering of OCD-relevant behaviours in Sapap3-KOs. **A)** A three-cluster solution revealed 2 clusters with elevated grooming, and one with low grooming. **B)** PPI was impaired in the first high-grooming cluster, but intact in the other high-grooming cluster. The third cluster showed normal behavior in both paradigms. * indicates significant Bonferroni post-hoc test comparing clusters *** $p<0.001$, **** $p<0.0001$. n=18 KO mice.

Longitudinal progression of changes in grooming in Sapap3-KO mice

Individual trajectories for grooming across 2-8 months of age in Sapap3-KOs and WTs revealed that while WT grooming remains stable across time, progressively grooming levels increase in a growing subset of KO mice (Figure S3A-B). Importantly, this analysis shows that it is very rare for grooming to substantially decrease in KO mice once it has become elevated. When separating grooming at later ages (6-8 months) by lesion status, this shows that grooming is increased in KO-L relative to WT and KO-NL, and KO-NL relative to WT (Figure S3C-E).

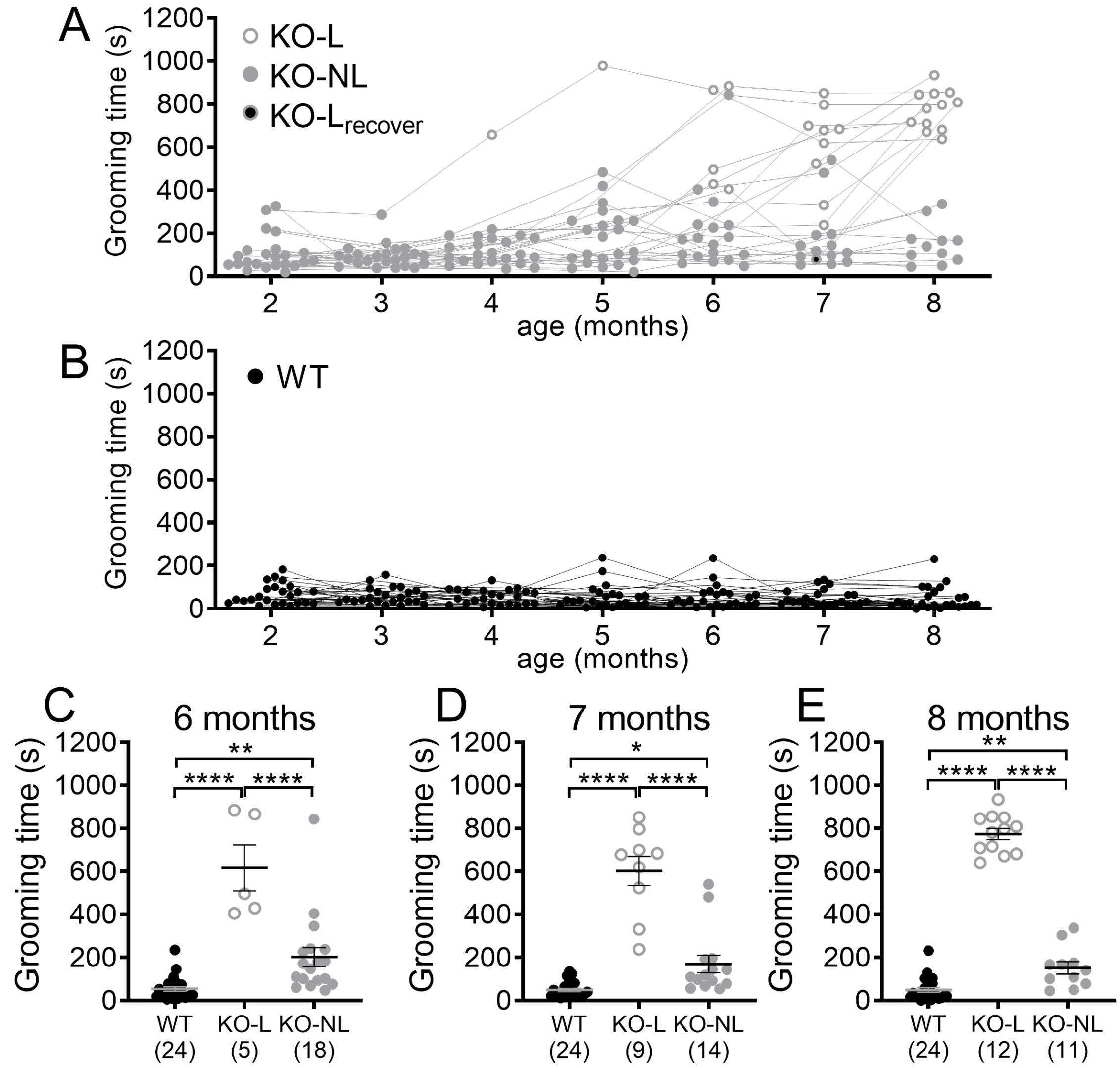

Figure S3: Longitudinal progression of grooming phenotype. **A)** Grooming trajectories for individual Sapap3-KO mice across the longitudinal study is plotted, showing a single instance when a KO-L mouse recovered to be re-classified to KO-NL in a subsequent month (designated KO-$L_{recover}$, it was later classified to KO-L at 8 months of age). **B)** Grooming trajectories for WT mice show no substantial changes over the same period. Grooming was increased in KO-L relative to WT and KO-NL, and KO-NL relative to WT, from 6-8 months of age **(C-E)**. * indicates results of Bonferroni post-hoc test, * $p<0.05$, ** $p<0.01$, **** $p<0.0001$. n=24 WT, 23 KO. KO= knockout, KO-L= KOs with lesions, KO-NL= KOs without lesions, WT= wild-type.

Early grooming phenotype

Assessment of post-weaning grooming phenotype from 3-8 weeks of age revealed a significant age dependent difference in grooming in Sapap3-KOs (Figure S4; age x genotype interaction, $F_{(5,160)}$=8.5, p<0.0001). Post-hoc tests revealed that grooming was increased in WT relative to KO at 3 weeks of age, whereas grooming was increased in KO relative to WT at 7-8 weeks of age.

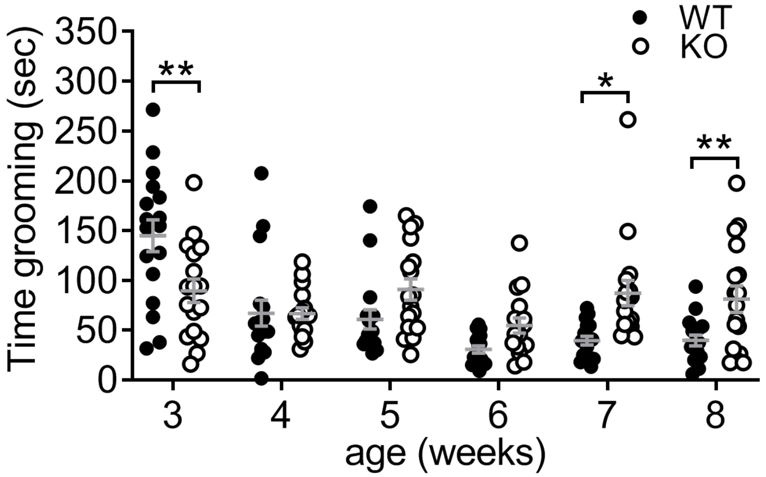

Figure S4. Early progression of grooming phenotype in Sapap3-KO mice. The early progression of the grooming phenotype was examined during the first month of life after weaning (3-8 weeks of age). Data from 8 weeks has been published elsewhere[1]. There were time-dependent differences in grooming between the genotypes. Post-hoc tests demonstrated that WTs showed elevated grooming at 3 weeks of age relative to KOs ($t_{(192)}$=3.8, p=0.0011), whereas at 7 and 8 weeks of age grooming was elevated in Sapap3-KOs (7 weeks, $t_{(192)}$=3.3, p=0.007; 8 weeks, $t_{(192)}$=2.9, p=0.029). * indicates results of Bonferroni post-hoc test, * p<0.05, **p<0.01. n=17 WT (9 males), 15 KO (7 males). KO = knockout, WT= wild-type mice.

Longitudinal progression of changes in PPI and startle in Sapap3-KO mice,

PPI significantly increased across 2-8 months of age in both groups (Figure S5A; $F_{(6, 270)}$=29.2, p<0.0001), and there was a trend for a reduction in PPI in Sapap3-KOs compared to WTs across 2-8 months of age (p=0.095). However, there were significant age-dependent

differences in startle between the genotypes (Figure S5B; interaction p=0.001), with Sapap3-KOs showing reduced startle relative to WT between 2-5 months of age. Therefore, PPI could only be reliably compared between WT, KO-NL, and KO-L between 6-8 months of age when startle was comparable between the groups (Figure S5C-E; one-way ANOVA comparing groups: 6 months p=0.13, 7 months p=0.65, 8 months p=0.18).

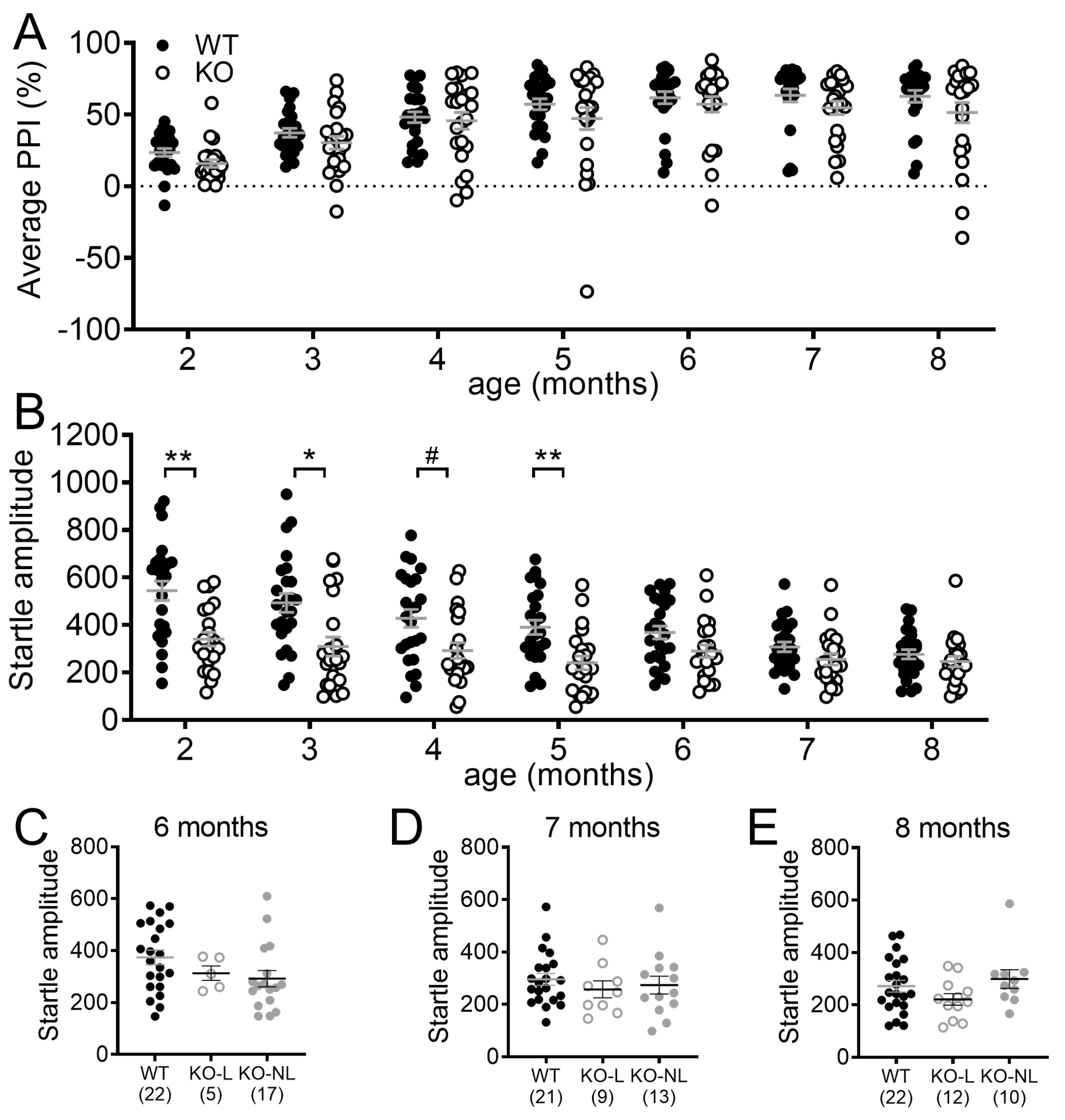

Figure S5: Longitudinal progression of changes in PPI, startle and grooming in Sapap3-KO mice. **A)** PPI significantly increased across 2-8 months of age in both groups. There was a trend for a reduction in PPI in Sapap3-KOs compared to WTs across 2-8 months of age (p=0.095). **B)** There were significant age-dependent differences in startle between the genotypes (interaction p=0.001), with Sapap3-KOs showing reduced startle relative to WT from 2-5 months of age. Therefore, PPI could only be reliably compared between WT, KO-NL and KO-L from 6-8 months of age when startle was comparable between the groups. Importantly, when KOs were separated into KO-L and KO-NL, startle was not significantly different from WT between 6-8 months of age **(C-E)**. * indicates results of Bonferroni post-hoc test, # 0.05<p<0.10, * p<0.05, ** p<0.01. Note, for panels A,B all mice are included (n=24 WT, 23 KO), whereas for panels C-E mice excluded from PPI analysis have been removed, resulting in different group sizes. KO= knockout, KO-L= KOs with lesions, KO-NL= KOs without lesions, WT= wild-type, PPI= prepulse inhibition.

Relationship between grooming and PPI in longitudinal cohort

PPI was significantly correlated with grooming at 8 months of age (Figure S6C; R=-0.59, p=0.0036), but not 6-7 months of age (Figure S6A-B; 7 months: R=-0.12, 6 months: R=0.09). Unbiased cluserting on data from 8 month old mice replicated findings from Figure S2, revealing 3 KO sub-types that differed in grooming and PPI (Figure S7A-B; grooming: one-way ANOVA, $F_{(2, 19)}$=137.2, p<0.0001; PPI: one-way ANOVA, $F_{(2, 19)}$=42.8, p<0.0001). For this cohort, all lesioned animals were classified to cluster 1 (n=6) and 2 (n=6) and all non-lesioned animals were classified to cluster 3 (n=10).

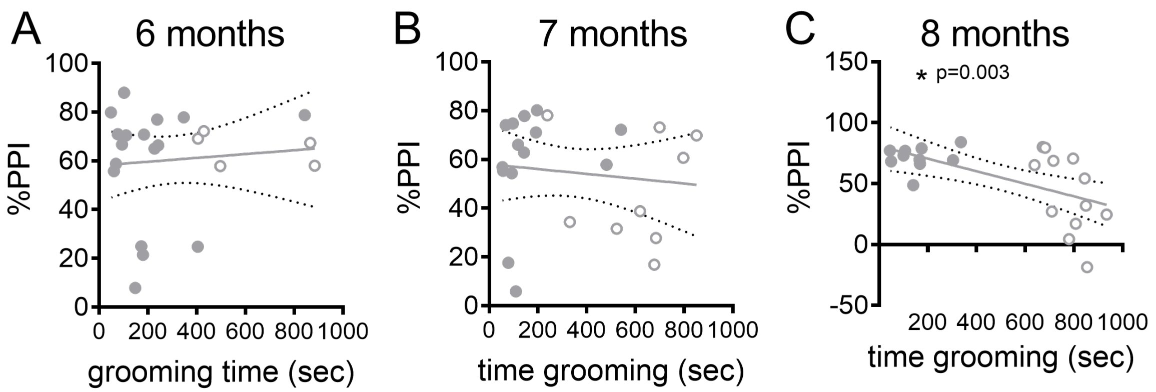

Figure S6: Correlation between PPI and grooming in longitudinal cohort. **A-B)** At 6 months of age, PPI was not correlated with grooming in Sapap3-KOs. **C)** At 8 months of age, PPI was negatively correlated with grooming, similar to Figure 1C (independent cohort). n=22 KO mice.

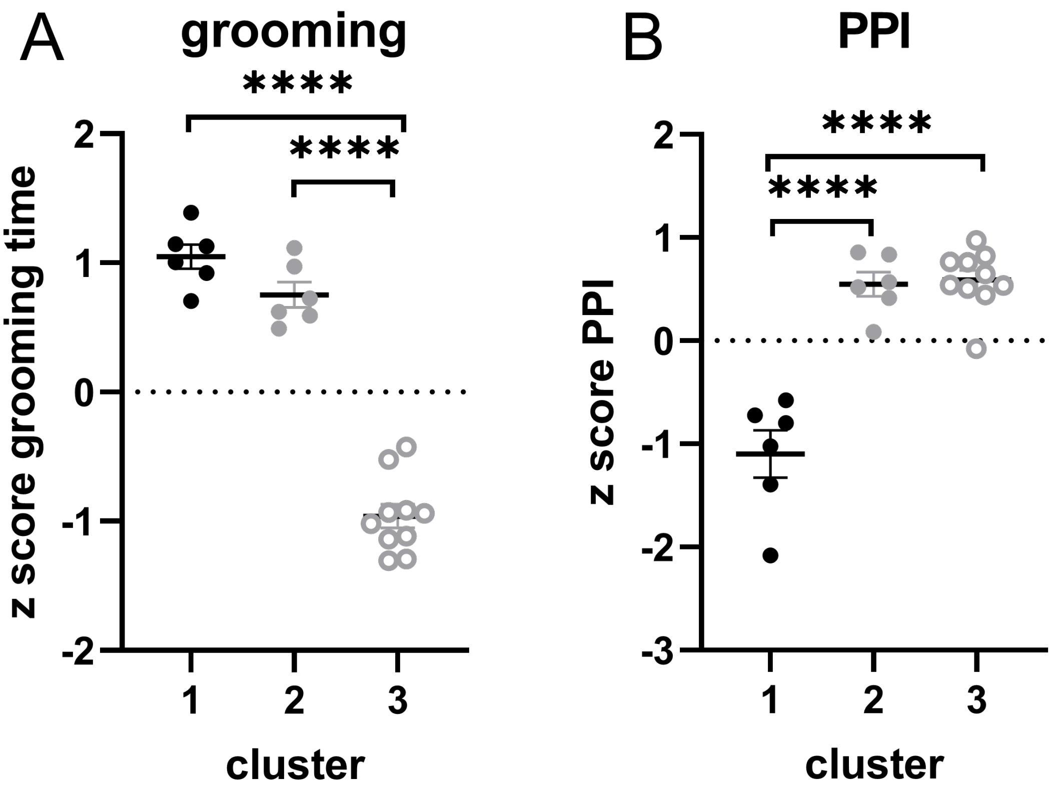

Figure S7: Unbiased clustering of OCD-relevant behaviours in Sapap3-KOs from 8 months age timepoint in longitudinal cohort. **A)** Grooming was significantly increased in cluster 1 and 2 relative to cluster 3. **B)** PPI was significantly impaired in cluster 1 relative to clusters 2 and 3. . * indicates significant Bonferroni post-hoc test comparing clusters, **** p<0.0001. n=22 KO mice.

Representative images of total binding and non-specific binding sections for autoradiography assays

Receptor binding was calculated by measuring luminosity in striatal regions of interest (Figure S8B) on total binding (TB) and nonspecific binding (NSB) slides (Figure S8A for representative images). These values were then converted to radioactivity (uCi/g) using a tritium standard curve, and NSB radioactivity was subtracted from TB radioactivity to give specific receptor/transporter binding value.

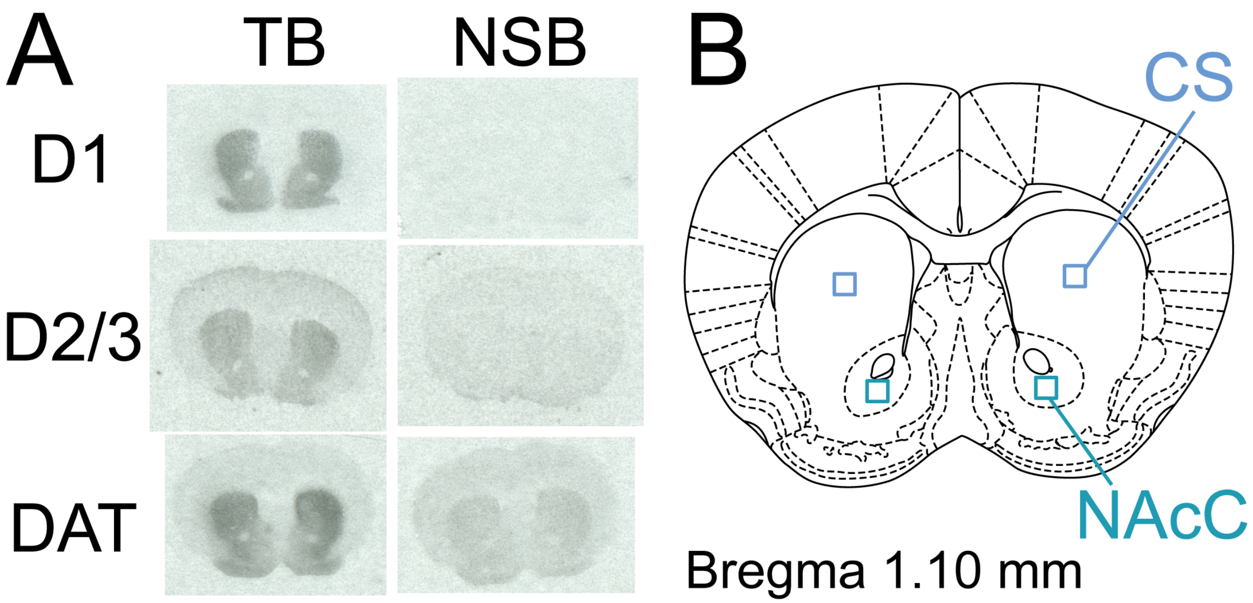

Figure S8. Representative autoradiography images and regions of interest. **A)** Representative TB and adjacent NSB autoradiographs from striatum for D1 receptor, D2/3 receptor, and DAT binding. **B)** Schematic diagram of coronal section with positioning of regions of interest used for quantification. CS= central striatum, NAcC= Nucleus accumbens core, DAT= dopamine transporter, TB= total binding, NSB= non-specific binding.

No sex differences in disruption of PPI or compulsive grooming in Sapap3-KO mice

Male and female mice were used in experiment 3. The data from these studies were used to investigate sex differences, given recent observations that PPI was disrupted in female OCD patients relative to healthy controls, but not in males[2]. In experiment 3, at 8 months of age there were no effects of sex (Figure S9A, main effect of group $F_{(2,38)}$=6.6, p=0.003, main effect of sex, p=0.71, sex x group interaction p=0.82). Planned Bonferroni post-hoc tests were performed to compare the groups in each sex separately, and although this analysis was underpowered it supported disruption of PPI in KO-L of both sexes (Figure S9A: male KO-L vs WT, $t_{(38)}$=2.2, p=0.0921, KO-L vs KO-NL $t_{(38)}$=2.5, p=0.045; female KO-L vs WT, $t_{(38)}$=2.5, p=0.05; for all other comparisons p>0.20). Severity of compulsive grooming phenotype also did not differ between the sexes (Figure S9B; main effect of group, p<0.0001; main effect of sex, p=0.948, interaction p=0.509).

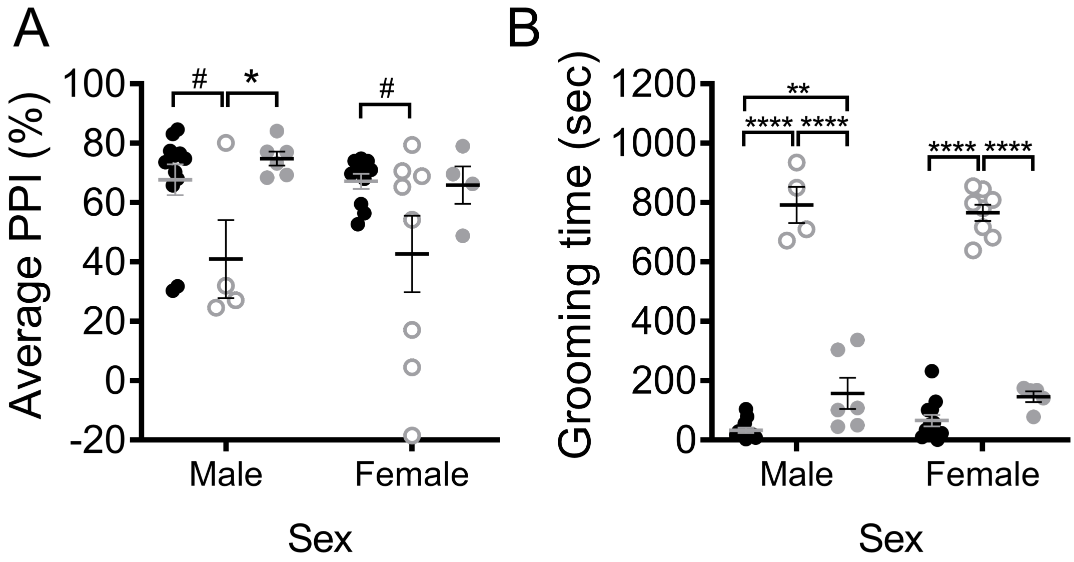

Figure S9. No sex differences in disruption of PPI or compulsive grooming in Sapap3-KO mice. **A)** Data from 8 month old mice from the longitudinal testing cohort (experiment 3) showed no

evidence for sex differences in disruption of PPI. **B)** Compulsive grooming severity from experiment 3 also showed no evidence for sex differences. * indicates significant Bonferroni post-hoc test. # 0.1>p>0.05; * p<0.05,** p<0.01, ****p<0.0001. male: n=4 KO-L, 6KO-NL, 12 WT; female: n= 8 KO-L, 4 KO-NL, 10 WT. KO-L= KO mice with lesions, KO-NL= knockout mice without lesions, WT= wild-type, PPI= prepulse inhibition.

Discussion

Detection of early changes in PPI and grooming in KOs

PPI could not be reliably compared between Sapap3-KOs and WT at early ages due to differences in startle amplitude. Therefore future studies may seek to “titrate” startle intensity between the groups (i.e. use lower intensity acoustic stimulus to induce startle in WT mice) so that startle is similar between the groups, allowing for more reliable comparison of PPI at earlier ages during the emergence of compulsive grooming in Sapap3-KOs[3].

In the longitudinal study (experiment 3), we were unable to replicate the findings of experiment 4 demonstrating an increase in grooming in Sapap3-KO mice at 8 weeks/2 months of age. Instead, we first saw grooming-related genotype differences emerge at 4 months of age. Detection of the earliest, mildest grooming phenotype may therefore be dependent on regular testing and handling, which may be the cause of lower variance and lower mean levels of grooming in WT control mice in experiment 4 (weekly testing during experiment 4: 8 weeks of age WT mean = 39.7, SD= 22.5; monthly testing during experiment 3: 2 months of age WT mean= 67.0, SD= 47.4).

Table S1: reagents and conditions used for autoradiography

		Ligand concentrations		Binding conditions					
Receptor	Ligand	M	Ci/mmol	Temp	Time	Inhibitors	Buffer	NSB	Exposure time
D1	$[^3H]$-SCH 23390[1]	2nM	81.9	RT	1 hr	1µM Ketanserin[2]	50mM Tris-HCl[2], 1mM $MgCl_2$[2], 120mM NaCl[2], 2mM $CaCl_2$[2], 5mM KCl[2]	10µM cis-flupenthixol[2]	3.5 weeks
D2/3	$[^3H]$-Raclopride[1]	5nM	73.8	RT	1.5 hr	None	Same as D1	10µM Haloperidol[2]	7.5 weeks
DAT	$[^3H]$-GBR 12935[1]	2nM	41.3	4ºC	20 hrs	1µM cis-Flupenthixol[2]	50mM HNa_2PO_4[2], 70mM NaCl[2], 0.025% BSA[2]	10µM Mazindol[2]	3 weeks

Superscript indicates chemical manufacturer, with [1]= Perkin Elmer (Boston, MA), [2]= Sigma Aldrich (St Louis, MO), BSA= Bovine Serum Albumin, RT = Room temperature (~25ºC), DAT= dopamine transporter.

References

1 Corbit, V. L., Manning, E. E., Gittis, A. H. & Ahmari, S. E. Strengthened Inputs from Secondary Motor Cortex to Striatum in a Mouse Model of Compulsive Behavior. *The Journal of neuroscience : the official journal of the Society for Neuroscience* **39**, 2965-2975, doi:10.1523/JNEUROSCI.1728-18.2018 (2019).

2 Steinman, S. A. *et al.* Prepulse Inhibition Deficits Only in Females with Obsessive-Compulsive Disorder. *Depression and anxiety* **33**, 238-246, doi:10.1002/da.22474 (2016).

3 Young, J. W., Wallace, C. K., Geyer, M. A. & Risbrough, V. B. Age-associated improvements in cross-modal prepulse inhibition in mice. *Behavioral neuroscience* **124**, 133-140, doi:10.1037/a0018462 (2010).
